# Supplementary material for: Modulation of Primary Cilia by Alvocidib Inhibition of CILK1
Source: Int J Mol Sci. 2022 Jul 23;23(15):8121. doi: 10.3390/ijms23158121 (PMC9329819; doi:10.3390/ijms23158121)
Supplement: Supplementary file 1 [file ijms-23-08121-s001.zip › ijms-1794897-supplementary.pdf]

Supplementary material

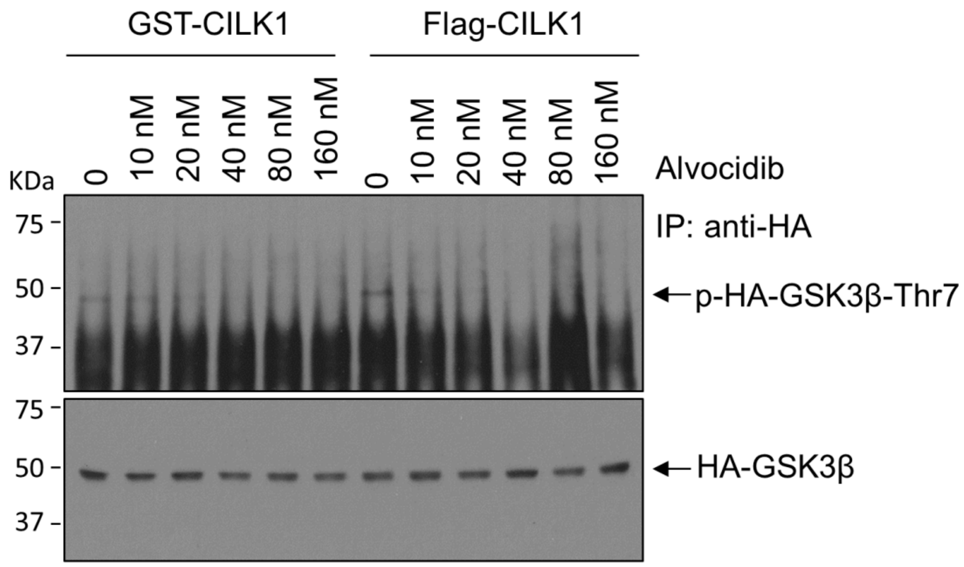

Figure S1. Alvocidib effect on CILK1 phosphorylation of GSK3β in cells. GST-CILK1 or Flag-CILK1 was co-transfected with HA-GSK3β (substrate) into HEK293T cells. Forty-eight hours after transfection, cells were treated with increasing concentrations of Alvocidib in DMSO for 15 mins before lysis. HA-GSK3β was immunoprecipitated from cell extracts with mouse monoclonal HA-tag antibody (12CA5) and Western blotted for total and phospho-T7 HA-GSK3β signals. .
